# The Impacts of Engagement in Men's Sheds on Incidental Physical Activity and Wellbeing Outcomes

**DOI:** 10.1002/hpja.958

**Published:** 2025-01-07

**Authors:** Briana Guerrini, James J. Clarke, Brendan J. Smith, Joanne A. McVeigh, Kirsten Holmes, James Wild, Rebecca Talbot, Jaxon Ashley, Peter M. McEvoy

**Affiliations:** ^1^ School of Population Health Curtin University Perth Western Australia Australia; ^2^ enAble Institute Curtin University Perth Western Australia Australia; ^3^ School of Allied Health Curtin University Perth Western Australia Australia; ^4^ School of Physiology University of Witwatersrand Johannesburg Gauteng South Africa; ^5^ School of Management and Marketing Curtin University Perth Western Australia Australia; ^6^ Men's Sheds of Western Australia Perth Western Australia Australia; ^7^ Centre for Clinical Interventions North Metropolitan Health Service Perth Western Australia Australia

**Keywords:** accelerometer, community organisation, men's sheds, men's wellbeing, physical activity, sedentary behaviour

## Abstract

**Background:**

As a large proportion of older adults are insufficiently active, it is imperative to identify ways to increase incidental physical activity. Men's Sheds, a mutual‐aid, community‐based organisation appear to be a promising approach for optimising wellbeing outcomes.

**Objectives:**

To investigate whether Men's Sheds attendance is associated with higher levels of physical activity, and the relationships between physical activity, health‐related quality of life (HRQOL), and wellbeing in Men's Shed members.

**Methods:**

Participants (*N* = 45) wore a hip accelerometer (Actigraph GTX‐9) for 11 days. The majority (*n* = 30, *M*_age_ = 72.3 ± 9.4) also consented to complete an online questionnaire investigating HRQOL and wellbeing.

**Results:**

Linear mixed models revealed members, on average, spent an additional 34 min in light physical activity, had an extra six breaks in sedentary behaviour, and took an extra 1193 steps on days they attended Men's Sheds, though, these effects were small. Physical activity was not significantly associated with HRQOL and wellbeing.

**Conclusion:**

Men's Sheds appear to be a valuable approach for increasing light intensity physical activity, breaks in sedentary behaviours, and step count in older adults. However, additional Men's Shed activities specifically targeting increased movement may be required to realise greater impacts on health‐related quality of life and wellbeing.

**So What?:**

Men's Sheds may provide a valuable opportunity to increase some indices of physical activity, which may contribute to better overall health. While these effects may be small for active Men's Shed members, these effects may be more appreciable for more sedentary individuals.

## 1 Introduction

Identifying ways to promote incidental physical activity in older adults is vital as individuals generally become less active with age [1]. Increasing age heightens the risk for developing chronic conditions and disease, reduced physical function, and depression [2]. Consequently, research and public policy are increasingly focused on healthy ageing—developing and sustaining functional capacity in older age that promotes wellbeing [3]. Implementing practical ways of addressing determinants of health‐related quality of life (HRQOL) and wellbeing to promote healthy ageing is therefore a public health imperative.

Regular physical activity is a well‐established predictor of HRQOL and wellbeing [4, 5]. Physical activity is any voluntary movement (i.e., activities of daily living, active transportation, leisure activity, and exercise) that expends energy [6]. Increasing physical activity in older adults is important as insufficient physical activity is one risk factor associated with all‐cause mortality and increased morbidity [4].

Sedentary behaviour is associated with unfavourable health outcomes (e.g., increased risk of non‐communicable diseases and all‐cause mortality) [7] and limits engagement in physical activity [8]. Sedentary behaviour refers to behaviours (i.e., lying down, reclining, or sitting) where energy expenditure is low (≤ 1.5 metabolic equivalents [METs]) [9]. While most research has focused on the volume of sedentary behaviour (i.e., time spent in sedentary behaviour) [10], breaks in sedentary behaviour may be uniquely associated with health outcomes [11]. If breaks in sedentary behaviour are associated with HRQOL and wellbeing, they may become a feasible target to improve wellbeing outcomes in older adults.

Understanding facilitators and barriers to physical activity for particular populations can guide more targeted health promotion efforts. While gender‐sensitive sports‐based interventions appear to be valuable for optimising men's physical activity engagement (e.g., Kwasnicka et al. [12]), the majority of participants in such interventions are below the age of 65 (see Sharp et al. [13]). Older men may experience diverse limitations that hinder participation in physical activity programs such as traditional gender norms, programs being too narrow in scope, and physical limitations such as specific ailments, ageing related aches and pains, risk of injury, fear of falling, and increased recovery time [14, 15]. Therefore, community organisations that promote incidental low‐intensity exercise may be more successful in engaging older men in programs that promote physical activity.

## 2 Men's Sheds as a Means to Increase Physical Activity in Older Men

Men's Sheds is one community‐based, mutual‐aid, health promotion initiative that may indirectly increase physical activity, HRQOL, and wellbeing outcomes in older men. Men's Sheds are safe places for men to congregate, socialise, and participate in shared activities. Men's Sheds are inclusive spaces that welcome all males; however, some Men's Sheds permit women as members, though this choice is contingent upon the membership at each Men's Shed site [16]. Although some empirical support exists for Men's Sheds' positive impacts on wellbeing, the preponderance of such evidence focuses on social factors [17]. The facilitators and recommendations for optimising older men's engagement in health promotion initiatives [15] are embedded within the Men's Shed movement, supporting Men's Sheds' health enhancing capacities.

Qualitative evidence suggests that participation in practical Men's Shed activities such as gardening, woodwork, and metalwork, can increase members' physical activity and reduce sedentary behaviours [18, 19]. While only one study has assessed self‐report physical activity in Men's Shed members [20] there are no behavioural investigations of physical activity in this context. This is particularly important because self‐report physical activity data do not always correspond to device‐measured physical activity [21].

## 3 The Current Study

While a small body of evidence has investigated physical activity in Men's Sheds, prior research is largely qualitative and relies on subjective perceptions of physical activity. Investigating the relationships between Men's Shed members' physical activity and health and wellbeing outcomes will extend our understanding of whether Men's Sheds are a feasible means for increasing incidental physical activity in older adults to improve their health. We aimed to investigate whether Men's Shed attendance was associated with higher levels of physical activity, and whether physical activity in this cohort was associated with HRQOL and wellbeing. The first hypothesis (H1) was that members will engage in more physical activity and breaks in sedentary behaviour on days they attend Men's Sheds compared to non‐Men's Shed days. The second hypothesis (H2) was that accelerometry‐based physical activity assessments will be positively associated with HRQOL and wellbeing.

## 4 Methods

### 4.1 Participants

Convenience sampling was used via advertising promotional study flyers on Men's Shed websites, social media, and emails to Men's Shed leaders. Inclusion required participants to provide informed consent, be a current member of a Men's Shed in Western Australia, and English proficiency. In total, 332 individuals completed the online questionnaire. Of those individuals who completed the online questionnaire, 30 participants provided consent to wear an Actigraph GTX‐9 accelerometer device on their hip for 11 days. As a result, self‐report data including demographic information is only available for these 30 participants who participated in the online questionnaire. These 30 participants comprised the sample for H2. An additional 15 participants who did not provide consent to participate in the online survey provided consent to wearing the accelerometer, resulting in a final sample size of 45 individuals who wore the accelerometer. These 45 participants comprised the sample for H1.

### 4.2 Measures

#### 4.2.1 Health‐Related Quality of Life

HRQOL was measured using utility scores calculated from the 5‐item EuroQol—5 Dimension scale (EQ‐5D‐5L scale) [22]. Participants rated the amount of difficulty they experienced across five domains (mobility, self‐care, usual activities, pain/discomfort, and anxiety depression) that comprised one item per domain, on a 5‐point scale ranging from 1 (*no problems*) to 5 (*unable to*). Utility scores generally range from 0 (death) to 1 (perfect health) and were calculated by applying a tariff reported by Norman et al. [23] to these health states. The EQ‐5D‐5L has good psychometric properties [24]. Raw scores had acceptable internal consistency in our sample (*α* = 0.68) and were highly correlated with utility scores (*r* = 0.94, *p* < 0.001).

#### 4.2.2 Wellbeing

The World Health Organisation‐Five Well‐being Index (WHO‐5 Index) [25] was used to assess mental wellbeing on a 6‐point Likert scale ranging from 0 (*at no time*) to 5 (*all the time*). Raw scores are summed and multiplied by four, with total scores ranging from 0 (worst possible wellbeing) to 100 (highest possible wellbeing). The WHO‐5 has adequate psychometric properties [26] and had good internal consistency in our sample (*α* = 0.89).

#### 4.2.3 Physical Activity

Participants were asked to wear the Actigraph GTX‐9 accelerometer device (Actigraph LLC, Pensacola, FL, USA) on their right hip for 11 days, which recorded data at a frequency of 30 Hz, and data were collapsed into 60‐s epochs. Accelerometer data were processed in SAS (Version 9.3, SAS Institute, Cary, NC, USA) using a validated semi‐automatic algorithm [27]. During the processing, the algorithm separated waking wear physical activity data from non‐wear or sleep data. Common cut points were used to classify each minute as sedentary (< 100 counts per minute [cpm]), light intensity (100–1951 cpm), moderate intensity (1952–5724 cpm), or vigorous intensity (> 5724 cpm) [28, 29].

Physical activity measures used in this study were time spent in physical activity intensities (light, moderate, and vigorous), step count, sedentary minutes, and sedentary breaks [27]. A break in sedentary behaviour was defined as movement (≥ 100 cpm) for at least 1 min after a sedentary bout.

### 4.3 Procedure

Ethical approval was obtained from Curtin University's Human Research Ethics Committee [HREC2022‐0148]. The survey was disseminated to members of Men's Sheds in Western Australia via email. See McEvoy et al. [20] for more detail regarding the procedure and content of this survey. Participants were informed that participation put them in the draw to win one of 10 $50 AUD vouchers to a hardware store. Survey data were collected between April 2022 and July 2022.

Participants who agreed to wear the accelerometer devices were provided with instructions for wearing the accelerometer, which also included the contact details of a researcher in case members had questions about the device. The display on the accelerometers were prepared as blank so that participants could not view their physical activity metrics. Accelerometer data were collected between May 2022 and August 2022.

### 4.4 Data Analysis

Analyses were conducted using Jamovi [30]. The first hypothesis was tested using linear mixed models, which compared levels of physical activity on Men's Shed versus non‐Men's Shed days (fixed effect), and a random intercept for participants was included to account scores being correlated within individuals. Two extreme outliers in step count were identified for one individual on consecutive days (47 569 and 66 996). These variables were truncated to be within ±3.29 SD of the mean [31]. The analysis was run with these outliers truncated and removed. Cohen's d was calculated to measure the size of the effect, and effect sizes were interpreted as small (*d* = 0.2), medium (*d* = 0.5), or large (*d* = 0.8) [32].

The second hypothesis was tested using Pearson's bivariate correlations for daily average scores, where coefficients were interpreted as small (*r* = 0.10), medium (*r* = 0.30), or large (*r* = 0.50) [32]. Prior to analysis, univariate outliers were identified for daily average moderate activity (*n* = 1), vigorous activity (*n* = 1), and step count (*n* = 1). These three outliers were truncated to be within ±3.29 SD of the mean [31]. To further improve the normality of these outliers, a square root and log transformation were performed [31]. The remaining data were approximately normal based on skewness and kurtosis (*z*‐values ±1.96) [33]. Correlations were run with the truncated and transformed variables but as the pattern of results were identical, only the results with the raw data are reported.

## 5 Results

### 5.1 Descriptive Statistics

Descriptives were only available for participants with actigraphy data (*N* = 45) who also consented to provide self‐report data (*N* = 30; *n* = 29 men, *n* = 1 woman). Therefore, self‐report descriptives are only available for 30 participants (see Table 1).

**TABLE 1:** Descriptive statistics.

Variable	*M*	SD	*N*
Age (range 39–86, years)	72.3	9.4	30
HRQOL	0.78	0.18	30
Wellbeing	62.0	24.5	30
Number of days members attended the Men's Shed	2.0	1.6	45
Number of days members did not attend the Men's Shed	3.3	2.2	45
Number of days of accelerometer wear	5	3	45
Accelerometer wear per day (min)	778	88	45
Time spent in light activity per day (min)	274	88	45
Time spent in moderate activity per day (min)	18	23	45
Time spent in vigorous activity per day (min)	< 1	1	45
Time spent in sedentary behaviours (min)	487	83	45
Number of breaks in sedentary behaviour per day	69	15	45
Number of steps per day (Mdn = 11 652)	12 255	4446	45

### 5.2 Association Between Physical Activity and Men's Shed Visits

To test the first hypothesis, linear mixed models comparing physical activity on Men's Shed vs. non‐Men's Shed days were run. Models with raw data are reported except where truncating or removing outliers impacted on the pattern of results. Linear mixed models revealed that there was a significant main effect of Men's Shed Days for light activity, number of breaks in sedentary behaviour, and number of steps. Men's Shed days (vs. non‐Men's Shed days) were associated with 34 additional min of light activity and six more breaks in sedentary behaviour and had the largest effects (see Table 2). The model for steps per day fell short of statistical significance, even when two extreme outliers were truncated (*p* = 0.06), despite Men's Sheds days being associated with an additional 1111 steps compared to non‐Men's Shed days. When the two outliers were removed (two consecutive days for one individual of 47 569 and 66 996 steps, next highest value in the sample = 26 758), then the additional steps on Men's Sheds days rose to 1193 and the effect was statistically significant (*p* < 0.05), however the confidence intervals remained wide and this effect was still small. Men's Shed days were also associated with spending 14 fewer minutes in sedentary behaviour, three additional minutes in moderate activity, and low levels of vigorous activity, but these differences were not statistically significant, effect sizes were small, and confidence intervals were wide.

**TABLE 2:** Linear mixed models comparing Men's Sheds days to non‐Men's Sheds days.

Dependent variable	Men's Shed day mean (SE)	Non‐Men's Shed day Mean (SE)	Estimate (SE)	95% CI (LL, UL)	*p*	Cohen's (*d*)
Time sedentary (min)	480.7 (15.3)	494.8 (13.5)	−14.1 (14.5)	−42.50, 14.34	0.332	−0.15
Sedentary breaks (*n*)	75.3 (2.5)	69.4 (2.2)	5.9 (2.6)	0.89, 10.94	0.022	0.38
Light activity (min)	305.2 (13.7)	271.1 (12.5)	34.1 (11.2)	12.15, 56.13	0.003	0.39
Moderate activity (min)	20.1 (3.3)	17.1 (3.1)	3.0 (2.2)	−1.35, 7.31	0.178	0.14
Vigorous activity (min)	0.07 (0.02)	0.03 (0.03)	−0.03 (0.04)	−0.10, 0.04	0.370	−0.24
Steps (outliers truncated)	13 330 (671)	12 219 (732)	1111 (597)	−58.3, 2280	0.064	0.24
Steps (outliers removed)	13 107 (698)	11 914 (646)	1193 (541)	133, 2253	0.029	0.27

*Note:* Means are estimated marginal means. *N* = 45 participants. The two outliers were on two consecutive days by the same individual (47 569 and 66 996 steps). Cohen's *d* = (${\bar{x}}_{1}–{\bar{x}}_{2})/s\text{pooled}$. *S*_pooled_ = ([SE_1_ × √45] + [SE_2_ × √45])/2. A total of 149 non‐Shed days and 88 Shed days were recorded across all participants. Data were therefore provided across an average of 3.3 non‐Shed days and 2.0 Shed days per participant.

### 5.3 Association Between Physical Activity, HRQOL, and Wellbeing

To test the second hypothesis, Pearson's bivariate correlations between physical activity, HRQOL, and wellbeing were calculated. Pearson's bivariate correlation coefficients (*r*) indicated that accelerometry‐based physical activity was weakly and non‐significantly associated with HRQOL (*r*s = −0.18–0.20, *p*s = 0.30–0.88) and wellbeing (*r*s = −0.18–0.11, *p*s = 0.35–0.78). HRQOL was most strongly associated with vigorous physical activity and wellbeing was most strongly but inversely associated with moderate physical activity, though these associations were weak, non‐significant, and the confidence intervals for all physical activity correlations were wide. See Table 3 for the specific correlations between physical activity, HRQOL, and wellbeing.

**TABLE 3:** Correlations with confidence intervals among physical activity and outcome variables.

Measure	1	2	3	4	5	6	7	8
1. HRQOL	—							
2. Wellbeing	0.76*** [0.55, 0.88]	—						
3. Time spent in light activity (min)	−0.01 [−0.36, 0.36]	0.11 [−0.26, 0.46]	—					
4. Time spent in moderate activity (min)	−0.18 [−0.51, 0.20]	−0.18 [−0.51, 0.19]	0.19^a^ [−0.12, 0.45]	—				
5. Time spent in vigorous activity (min)	0.20 [−0.18, 0.52]	0.10 [−0.27, 0.44]	–0.16^a^ [−0.44, 0.14]	–0.01^a^ [−0.31, 0.28]	—			
6. Time spent in sedentary behaviours (min)	−0.09 [−0.43, 0.28]	−0.12 [−0.46, 0.25]	−0.42** ^,^ ^a^ [−0.64, −0.15]	–0.26^a^ [−0.51, 0.04]	0.15^a^ [−0.16, 0.42]	—		
7. Number of breaks in sedentary behaviours	0.05 [−0.32, 0.40]	0.08 [−0.29, 0.43]	0.67*** ^,^ ^a^ [0.48, 0.81]	–0.05^a^ [−0.34, 0.25]	0.07^a^ [−0.23, 0.36]	–0.12^a^ [−0.40, 0.18]	—	
8. Number of steps	−0.13 [−0.47, 0.24]	−0.01 [−0.37, 0.35]	0.72*** ^,^ ^a^ [0.54, 0.84]	0.47** ^,^ ^a^ [0.20, 0.67]	–0.14^a^ [−0.42, 0.16]	−0.41** ^,^ ^a^ [−0.63, −0.13]	0.37* ^,^ ^a^ [0.09, 0.60]	—

*Note:* The values in square brackets indicate the 95% confidence intervals for each correlation. *N* = 30 except.^a^

^a^ *N* = 45.

* *p* < 0.05.

** *p* < 0.01.

*** *p* < 001.

## 6 Discussion

We investigated whether Men's Shed attendance was associated with more physical activity, and whether physical activity is associated with members' HRQOL and wellbeing. Our hypothesis that members will engage in more physical activity and breaks in sedentary behaviour on days they attend Men's Sheds compared to non‐Men's Shed days was supported (H1), as members spent more time in low intensity physical activity, had more breaks in sedentary behaviours, and had a higher step count on days they attended the Men's Shed. However, our hypothesis that accelerometry‐based measures of physical activity will be positively associated with self‐reported HRQOL and wellbeing was not supported (H2).

Our findings support the contention that Men's Shed engagement may be a means to improve low‐intensity and incidental exercise. Finding more low‐intensity exercise, breaks in sedentary behaviour, and steps on Men's Shed days compliments previous qualitative research noting subjective perceptions that members are more mobile at Men's Sheds and are less sedentary as a result of participating in activities at Men's Sheds [18, 19]. While engaging in more physical activity was not associated with better self‐reported HRQOL or wellbeing in our sample, this increase in light physical activity, breaks in sedentary behaviour, and step count on days members attend the Men's Shed indicates that Men's Shed engagement may still have important implications for health status. For example, replacing 30 min of sedentary time per day with an equal amount of light or moderate‐to‐vigorous intensity exercise confers with greater physical health [34]. In addition, increasing step count is one means of increasing breaks in sedentary behaviour and is associated with a reduced risk of all‐cause mortality with this effect plateauing in older adults between 6000 and 8000 steps per day [35]. Therefore, finding Men's Shed members to be more physically active on days they attend the Men's Shed may contribute to improved physical health variables. This increase in light physical activity, steps, and breaks in sedentary behaviour may not have an appreciable effect in our already active cohort, which, may be a consequence of Men's Shed engagement itself. However, this observed increase in physical activity may have a disproportionately positive effect for individuals who are currently inactive compared to people who are already active. For example, replacing 30 min of sedentary behaviour with 30 min of light physical activity per day is associated with a significant reduction in mortality risk [36]. The evidence of an effect in our study may suggest that if people who are currently inactive were to join a Men's Shed, the magnitude of observed effects and associated health benefits may be more meaningful for these individuals.

Physical activity was not significantly associated with HRQOL and wellbeing. Evidence regarding the association between device‐assessed physical activity and HRQOL and wellbeing across the literature is mixed [34, 37, 38]. While our non‐significant correlations between the outcomes and time spent in sedentary behaviour supports prior findings [34, 38], in contrast to other research, we did not find a significant association between step count and the outcomes [38, 39]. While HRQOL and wellbeing in our sample is comparable to age‐matched individuals (HRQOL *M* = 0.78 vs. *M* = 0.87 [40]; wellbeing *M* = 62.0 vs. *M* = 70.8 [41]), our difference in findings may reflect limitations in our study design. For example, our lack of expected effects may be due to reduced statistical power to detect these associations evidenced by the wide confidence intervals and our small sample size. While non‐significant, the association between breaks in sedentary behaviour and HRQOL and wellbeing is a novel finding as the preponderance of research has investigated associations between breaks in sedentary time with physical function and cardiometabolic health [11, 42]. This may be an interesting avenue to further explore with larger samples. Our findings, while inconclusive, indicate that in the Men's Shed context, physical activity may not be a prominent pathway to improved HRQOL and wellbeing. As physical activity is not a focus of the Men's Shed movement, there may be other factors influencing HRQOL and wellbeing outcomes such as social network quality in Men's Sheds [43].

There are multiple possible explanations for our observed non‐significant associations between physical activity, self‐reported HRQOL, and wellbeing. First, the period of measurement may not have been representative of participants' broader lifestyle. For example, the accelerometer data captured participants' physical activity over an 11‐day period, however, if participants behaviour was not representative of their typical physical activity patterns year‐round, then their movement over this relatively short period of time should not impact their HRQOL. Second, other lifestyle or Men's Shed‐related social factors may be more critical for predicting HRQOL in this sample. Third, the amount of physical activity in our sample is different to larger community‐dwelling samples of older adults in Europe and the United States of America (men and women aged 70–75 years). For example, while average breaks in sedentary behaviour was similar (*M* = 69 vs. *M* = 72 [10]), on average, our sample spent significantly less time in sedentary behaviours (487 vs. 679 min [44]), significantly more time in light physical activity per day (4.6 vs. 2.7 h [37]) and had a higher step count (12 225 vs. 7638 [45]). While these samples are not directly comparable to our sample, they offer a valuable reference point for interpreting our findings. As our sample appears to be highly physically active we may be observing a ceiling effect, whereby we have only captured the top scores in the upper range and missed the variation in physical activity levels that are representative of the Men's Shed cohort. The failure to find evidence to support our hypotheses might also be due to selection bias as perhaps only the most physically active members opted to wear the accelerometers and complete the survey.

## 7 Limitations and Future Research Directions

While this study provides a novel contribution to the existing Men's Sheds' evidence base and is the first study to use behavioural‐assessed measures of physical activity in the Men's Shed context, some limitations must be considered. First, the correlational design precludes causal inferences and any increase in physical activity cannot be solely attributed to activities within the Men's Sheds. For example, this association may in part, be attributed to travel to and from Men's Sheds or other activities that members may engage in prior or post attending the Men's Shed. Future research should consider conducting a longitudinal design that measures HRQOL and wellbeing at different time points or a comparison group design with newer Men's Shed members to ascertain the impact that Men's Shed attendance and incidental physical activity has on health and wellbeing outcomes. Second, self‐selection may have occurred as our sample appeared to be highly physically active and may not be representative of the entire Men's Shed population, reducing the generalisability of findings as there may be an under‐representation of inactive members. The generalisability of findings is further limited by the small sample size. Future research should use purposive sampling to capture members who are less physically active to see if the hypothesised relationships arise when there is a larger sample with more heterogeneity.

Third, while accelerometers worn on the hip are the best single location to detect a range of daily activities [46], incidental physical activity from Men's Shed activities may not have been accurately collected. As waist‐mounted accelerometers do not detect upper limb movements, energy expenditure estimation may be inaccurate when individuals carry different loads of weight during activity [47]. This may be particularly important for the Men's Shed context as activities such as woodwork, metalwork, and repairs are offered in many Men's Sheds and members' presumably use their upper body to engage in such activities.

## 8 Implications

Despite our finding that individuals engage in significantly more steps and spend more time in light physical activity intensity on days they attend the Men's Shed, our results suggest this level of incremental engagement in incidental physical activity may not directly contribute to improved self‐reported HRQOL and wellbeing in the Men's Shed context. While we did not find an association between physical activity and wellbeing outcomes, this increase in physical activity may have a larger impact on unmeasured indices of physical health and wellbeing (e.g., blood sugar levels, heart health) and in other outcomes such as health service utilisation, which should be the focus of future research. Prior evidence has demonstrated an association between improved QOL and exercise interventions in older adults [48], so incorporating exercise activities may be associated with wellbeing outcomes in Men's Sheds, particularly for members who engage in low levels of physical activity. Additionally, it may be worthwhile to encourage Men's Shed members to join community groups targeted at increasing physical activity rather than incidental activity to optimise wellbeing outcomes. For physically active members, it may be more valuable for Men's Sheds to focus their efforts on the social environment of Men's Sheds and promote good quality relationships and psychological safety between members.

## 9 Conclusion

Men's Sheds membership is associated with enhanced wellbeing outcomes, however, the role of incidental physical activity in Men's Sheds is underexplored. This study sought to investigate how physical activity in Men's Shed members is associated with HRQOL and wellbeing. We found evidence that Men's Shed members spend more time in light physical activity, take more breaks in sedentary behaviours, and have a higher step count on days they attend the Men's Sheds compared to days they do not. While we did not find evidence supporting associations between accelerometer‐assessed physical activity, HRQOL, and wellbeing, it will be important for future research to extend our findings with larger samples by investigating relationships between physical activity and health outcomes, particularly in newer members, and the impact of dedicated exercise interventions targeting physically inactive members.

## Ethics Statement

Ethical approval was obtained from Curtin University Research Ethics Committee [HRE2022‐0148] and participants provided informed consent.

## Conflicts of Interest

James Wild, Jaxon Ashley, and Rebecca Talbot are employed by Men's Sheds of WA. While they contributed to the manuscript drafts, they did not have access to the raw data and did not conduct the analyses. The authors disclose no other potential conflicts of interest.

## Data Availability

Data are available from the corresponding author upon reasonable request. Due to ethical reasons, the data are not publicly available.
